# Smart Composite Hydrogels for Monitoring and Managing Chronic Wounds

**DOI:** 10.3390/gels12020120

**Published:** 2026-01-29

**Authors:** Jun Zhu, Yibin Huang, Junbo Tong, Antong Li, Bin Chu

**Affiliations:** School of Materials Science and Engineering, Xiamen University of Technology, Xiamen 361024, China

**Keywords:** composite hydrogel, intelligent dressing, multifunctional sensing

## Abstract

The precise management of chronic wounds poses a global medical challenge, owing to their complex and dynamically shifting pathological microenvironment, coupled with their inherent difficulty in healing. Traditional dressings, which lack capabilities for real-time monitoring and active intervention, fall short of meeting modern clinical needs. Composite hydrogels offer a novel solution to this problem. By integrating functional fillers within biocompatible hydrogel matrices, they form intelligent materials capable of sensing key wound parameters. This review systematically outlines the composite systems and material classification of such hydrogels designed for the intelligent monitoring of chronic wounds. It subsequently details the construction of multimodal monitoring systems and their applications across different types of chronic wounds., Finally, future development direction are discussed, aiming to advance the implementation of next generation, personalized intelligent wound management systems.

## 1. Introduction

With the aging of the global population and the increasing prevalence of metabolic diseases such as diabetes, the clinical management of chronic wounds, including diabetic foot ulcer (DFU) and pressure injuries has emerged as a serious medical challenge [1]. These wounds are characterized by a prolonged healing process, high susceptibility to infection and a complex pathological microenvironment [2]. Typically stalled in the inflammatory phase, they present a highly complex and dynamically shifting microenvironment, marked by persistent bacterial infection, excessive exudate, abnormal pH, and elevated levels of reactive oxygen species (ROS) [3,4]. Traditional dressings such as cotton or gauze provide only passive physical coverage, tend to adhere to the wound bed, and lack the capability for real-time monitoring of wound status, resulting in delayed optimal treatment [5,6]. Therefore, developing smart wound dressings capable of real-time sensing of physiological and pathological signals at the wound site and provide responsive feedback is essential for enabling precise diagnosis and treatment of chronic wounds [7].

Hydrogels, with their unique three-dimensional hydrophilic network structure, are considered ideal matrices because they mimic the natural extracellular matrix, possess high water content, and exhibit excellent biocompatibility [8]. They not only maintain a moist healing environment but also absorb exudate while allowing gas exchange to promote wound healing [9]. However, traditional single-component hydrogels exhibit significant limitations in practical applications. They typically lack electrical conductivity, signal transduction capabilities, and sufficient mechanical toughness, making it difficult to meet the stringent requirements for material sensitivity and stability demanded by smart sensing applications [10].

To overcome the performance limitations of single-component materials, the development of composite hydrogels has become a central strategy in current research on smart dressings [11]. Composite hydrogels are not simply physical mixtures of materials; instead, they are designed following function-oriented principles. Through physical blending, chemical cross-linking, or the construction of dual-network structures, an organic integration between the hydrogel matrix and functional fillers is achieved [12,13]. These fillers include a wide variety of materials, such as conductive polymers, carbon nanomaterials, and metal nanoparticles or metal-organic frameworks (MOFs), which can transmit electrical signals and often combine antibacterial properties with catalytic activity [14]. Via the synergistic “matrix + filler” effect, the resulting composite hydrogel exhibits enhanced mechanical strength and tissue adhesion. Furthermore, they gain the ability to convert chemical or physical signals from the wound microenvironment into quantifiable electrical or optical signals [15]. Nevertheless, selecting suitable composite materials and combination strategies to optimize hydrogel performance remain a major research challenge in this field, especially since real-world biomedical applications are highly complex and place extremely stringent demands on the properties of composite hydrogels [16].

**Figure 1:**
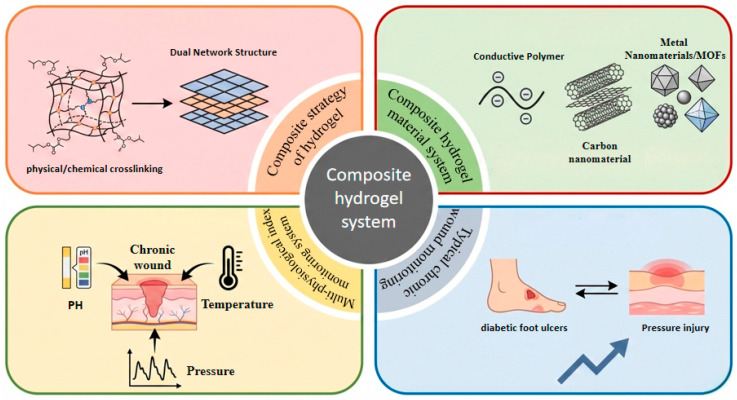
Composite hydrogel monitoring system: Construction strategy, material system, wound physiological monitoring and overview of typical clinical applications.

This review examines the design and clinical application of composite hydrogel dressings for the intelligent monitoring of chronic wounds (Figure 1). It begins by analyzing strategies for constructing high-performance composite hydrogels, covering physical and chemical cross-linking mechanisms as well as dual-network structure designs. The article then systematically surveys composite hydrogels across different material systems, including conductive polymers, carbon nanomaterials, metals, and MOF-based materials. Furthermore, It further elaborates on the mechanisms of action and applications of these dressings for monitoring key physiological indicators, such as wound pH, temperature, and pressure. Finally, by integrating two typical clinical cases—DFU and pressure injuries (PI)—the review assessess the development potential of smart composite hydrogels, discusses the remaining technical challenges, and suggests future research directions. The aim is to provide a theoretical foundation and a clear technical pathway for the development of next-generation intelligent wound-management systems.

## 2. Composite Strategy of Hydrogel

To achieve efficient and stable wound monitoring, the design of composite hydrogels is not merely a process of material stacking, but follows a clear function-oriented principle. Through straightforward and effective strategies that combine hydrogels with biocompatible functional components, wound dressings with broad-spectrum antibacterial and immunomodulatory properties can be developed, thereby promoting the healing of chronic wounds [17].

At the material selection level, the hydrogel matrix provides three-dimensional hydrophilic networks, mechanical support, and biocompatibility, while conductive components establish continuous charge-transport channels and convert chemical signals into electrical ones [10,18]. The pH, temperature and pressure-responsive units, on the other hand, serve as molecular probes that instantly detect local dynamic changes in the wound through their functional groups or structures. An ideal signal-response unit should exhibit high sensitivity, rapid response, good reproducibility and reversibility, as well as long-term stability [19]. Simultaneously, all materials must satisfy biocompatibility requirements. Natural polymer matrices or degradable synthetic materials are typically preferred to prevent foreign-body reactions at the wound site [20]. At the structural design level, it is essential to construct a network architecture that enables efficient signal transmission. First, the continuity and integrity of the hydrogel network must be ensured. By regulating the dispersion state of the signal-responsive components, a continuous pathway throughout the hydrogel can be established, guaranteeing that even subtle signal changes are effectively captured and transmitted. Secondly, the macroscopic structure should be designed to conform to the wound surface. Through chemical or physical cross-linking, the hydrogel can be endowed with suitable tensile strength and conformability, ensuring full contact between the sensing unit and the wound microenvironment [21,22].

### 2.1. Physical Crosslinking

Physical crosslinking forms a stable network between conductive components and the hydrogel matrix through non-covalent bonds such as hydrogen bonding, hydrophobic interactions, ion coordination, and π-π stacking [23]. This approach offers advantages including mild preparation conditions, no residual chemical reagents, and excellent biocompatibility, etc., to adapt to the biosafety requirements of chronic wound dressings. The core is to leverage the dynamic reversibility of non-covalent bonds to retain the flexibility and self-healing properties of hydrogels while constructing conductive networks [24].

The cross-linking network of physical cross-linked hydrogels is mainly constructed by a variety of dynamic non-covalent interactions, such as hydrogen bonds, ion coordination, host-guest recognition and ligand coordination. Because of non-covalent interactions, they exhibit reversibility, repairability, and responsiveness [25]. Among them, supramolecular hydrogel systems based on host-guest interactions have attracted significant attention due to their high dynamic reversibility and excellent selectivity. Xiong et al. [26] designed a supramolecular hydrogel system based on host-guest interactions of carboborane and β-cyclodextrin. This system mixes CB-modified dextran (DEX-CB) and β-CD-modified polyacrylic acid (PAA-CD) and rapidly forms a physical crosslinking network through strong, specific host-guest binding. In terms of ion coordination cross-linking, the combination of alginate and metal ions is a typical case, Li et al. [27] constructed a dual ion-crosslinked network, which not only markedly enhanced the material’s mechanical properties but also enabled programmable shape-changing behaviors, showcasing the potential of ion coordination in creating intelligent, responsive materials. Moreover, hydrogen bonding is extensively employed in constructing dynamically responsive hydrogels. For instance, Zhang et al. [28] designed a multi-responsive hydrogel by cross-linking polyethylene polyamines (PPAs) with gelatin via hydrogen bonds. Under acidic conditions, protonation of amine groups in PPA disrupts the hydrogen-bond network, leading to irreversible collapse of the gel structure.

The primary advantage of this crosslinking approach lies in the simple preparation and the dynamic nature of the resulting network. Mild molding conditions maximally retain the biological activity of the pH responsive units. The dynamically reversible crosslinking point confers the material rapid self-healing ability and excellent injectability, allowing it to perfectly fit irregular wounds [29]. Meanwhile, this looser cross-linked network provides an efficient channel for ion migration, enabling the composite hydrogel not only to achieve a higher gauge factor, but also to exhibit a shorter pH response time [30]. However, the nature of physical interactions also limits the use of such materials. The strength of non-covalent bonds is weak, resulting in generally poor mechanical properties of gels, which are prone to irreversible deformation or rupture under stress [31]. More critically, these dynamic interactions are highly susceptible to interference from the complex wound environment. Factors such as variations in ion types and concentrations within exudate, inflammatory cytokines, enzymes, and bacterial metabolites can all disrupt or weaken non-covalent interactions like hydrogen bonding, ion coordination, and host-guest recognition. This disruption causes network relaxation and dynamic imbalance of cross-linking points, leading to the gradual leaching of functional components such as conductive fillers and responsive units. The loss of these components not only degrades sensing performance—reducing conductivity and response sensitivity—but also induces baseline drift and diminished long-term stability. Consequently, the reliability and reproducibility of the data necessary for long-term, continuous clinical monitoring are significantly compromised.

### 2.2. Chemical Crosslinking

Chemical cross-linking establishes a stable three-dimensional network through the formation of covalent bonds between polymer chains. This approach imparts hydrogels with strong mechanical properties and swelling resistance, effectively meeting the stability needs of chronic wounds in long-term moist environments [32]. The key is to select a biocompatible crosslinker or use in situ polymerization to avoid wound irritation by residual chemicals [33].

The primary methods of chemical cross-linking include radical polymerization, click chemical and radiation crosslinking [34]. Radical polymerization cross-linking is initiated by blending conductive monomers with hydrogel monomers. For example, Gharachel et al. [35] developed a light-cured composite bioink for extrusion 3D bioprinting by compounding methacrylated alginate (MeALG) with human-derived bone allograft particles and the photoinitiator LAP. Their work demonstrates the significant potential of light-cured radical bioinks for constructing complex tissue-engineering scaffolds with activity-inducing functions. In another study, Blažic et al. [36] prepared porous Cel-g-PDMAEMA hydrogels with adjustable composition via radical polymerization, grafting poly (2-(dimethylamino)ethyl methacrylate) onto a cellulose backbone. These hydrogels maintained stable swelling behavior across acidic, neutral, and basic conditions.

The click-chemical cross-linking method offers high efficiency, good selectivity, broad tolerance to diverse chemical groups, and mild reaction conditions [37]. As an example, Saletti et al. [38] developed a click chemistry-based crosslinking strategy. A series of HA-FA-HEG-CL hydrogels were constructed by copper-catalyzed azide-alkyne cycloaddition of alkynyl hyaluronic acid grafted copolymers highly conjugated with double-ended azidated hexaethylene glycol. The cross-linking process is mild and selective, allowing the swelling behavior, rheological properties, and mesh size of the prepared hydrogel to be precisely tuned by varying the cross-linking density. In vitro cell studies further confirmed the good biocompatibility, offering a typical paradigm for click chemistry to construct controllable cross-linked hydrogel systems in the fields of drug delivery and tissue engineering. Cherri et al. [39] also constructed a redox-responsive hydrogel using click chemical crosslinking. In their system, acrylated hyperbranched polyglycerol and four-arm polyethylene glycol thiol served as cross-linking precursors, which undergo efficient chemically cross-linked under physiological conditions to form a three-dimensional network structure based on thioether bonds. Spectroscopic analysis confirmed the occurrence of the crosslinking reaction. This catalyst-free method proceeds under mild conditions, and the disulfide bonds incorporated into the gel network confer redox-responsive degradation behavior, enabling on-demand release of antimicrobial peptides. This study demonstrates the effectiveness and flexibility of chemical crosslinking in the construction of intelligent drug delivery systems.

Radiation cross-linking enables the preparation of soft, transparent hydrogels at room temperature within a short timeframe. Moreover, radiation exposure can serve as a sterilization step, eliminating the need for high-temperature autoclaving [40]. Relleve et al. [41] regulated the absorbed dose of gamma rays and polymer concentration to precisely control the cross-link density of carboxymethyl hyaluronic acid hydrogels. They achieved a tunable swelling rate ranging from 43 to 2400 g water/g dry gel, demonstrating successfully radiation-induced cross-linking of the hydrogel. Their study systematically examined the gel properties, swelling behavior, and biocompatibility of the resulting hydrogels, underscoring radiation cross-linking as a clean and efficient fabrication method.

This cross-linking approach provides hydrogel dressings with structural stability and programmable functionality, with its technological evolution progressing from the construction of static networks to the design of dynamic, intelligent response systems. Advanced cross-linking chemistry now serves as a key foundation for the next generation of intelligent dressings, whether it achieves the coupling of crosslink density and signal output with precise and controllable click chemistry fixed sensing units, with the help of dynamic covalent bonds, or achieves the in situ monitoring of physiological electrical signals through the integration of conductive networks. Looking ahead, to address the complex and variable chronic wound microenvironment, novel intelligent cross-linking strategies that integrate multiple response mechanisms, excellent biocompatibility and long-term signal stability will be the key to realize real-time and closed-loop management of wound status and finally promote the treatment of chronic wounds from passive care to active precise intervention.

### 2.3. Dual Network Structure

Dual network (DN) hydrogels have been proposed, inspired by the multilayered, energy-dissipating structure of biological tissues [42]. The core concept lies in constructing two interpenetrating polymer networks with distinct physical or chemical properties, typically combining a first network of high modulus and brittleness with the a second network that exhibits high toughness and extensibility, thereby achieving a complementary balance of stiffness and flexibility [43]. The toughening mechanism primarily relied on sacrificial bonds. In DN hydrogels, the first network is usually composed of highly cross-linked, rigid polymers, such as polyacrylamide, polyelectrolytes, as “sacrificial networks” that fractures preferentially to dissipate energy [44,45]. When the external force is applied, the chemical bond within the sacrificial network breaks first, dissipating substantial energy through a controlled fracture mechanism that prevent macroscopic crack propagation. The second network, typically composed of lightly cross-linked, soft polymers, such as sodium alginate or polyethylene oxide, forms a continuous elastic matrix [46]. As the network fractures, it acts as an elastic matrix to maintain the material’s integrity and redistribute stress, ultimately achieving a balance between high strength and high toughness. This structure design not only provides hydrogels with mechanical properties that match those of biological soft tissues [47] but also establishes a stable physical platform capable of supporting integrated functions such as sensing, conductivity, and drug release. Thus, dual-network architecture represents a key material strategy for developing high-performance intelligent wound dressings. Through a complementary rigid-flexible network design, interpenetrating network hydrogels achieve not only substantially enhance the strength and toughness, but also greatly improve its fatigue resistance and shape recovery capability under cyclic loading. These properties make them better suited to meet the long-term mechanical durability requirements of wearable sensors.

#### 2.3.1. Fully Chemical Crosslinked DN Hydrogels

In this type of cross-linked structure, both networks are covalently cross-linked. Lane et al. [48] reported a fully chemically cross-linked DN hydrogel constructed from microgel building blocks. Firstly, pH-responsive poly(ethyl acrylate-co-methacrylic acid) microgels were crosslinked via covalent bonds to form a rigid yet brittle first network. Subsequently, the swollen network initiates polymerization in a monomer solution containing acrylamide and a small amount of cross-linker, forming a flexible second network in situ. This unique network structure allows the first microgel network, acting as a reinforcing phase, to become highly swollen, and interpenetrated, while being constrained by the surrounding flexible second network, and finally synergistically achieves a significant synchronization between the material modulus and the fracture strain. Although such gels exhibit extremely high mechanical strength and structural stability, energy dissipation may cause irreversible damage to the covalent networks, resulting in limited self-healing capability [49].

#### 2.3.2. Physico-Chemical Crosslinked DN Hydrogels

To address the intrinsic lack of the self-healing in fully chemical cross-linked dual-network hydrogels, researchers have turned to physicochemical cross-linked DN systems that leverage reversible interactions. These designs combine dynamic physical cross-links, such as ionic bonds, hydrogen bonds, hydrophobic association, or host-guest interactions, as sacrificial bonds, with permanent chemical networks [49]. This approach endows the material with notable self-repair and fatigue resistance while preserving high mechanical strength.

Geng et al. [50] fabricated a directionally arranged CNF/MXene/Chitosan first network by interfacial polyelectrolyte composite spinning technique, then introduced a chemically cross-linked poly (acrylamide-co-acrylic acid) second network in situ, and then prepared a physicochemical mixed dual-network anisotropic conductive hydrogel by an in situ chemically cross-linked poly (acrylamide-co-acrylic acid) second network. In this asymmetric double network structure, the rigid first network is physically cross-linked and directionally arranged via electrostatic interactions and hydrogen bonds, while the flexible second network is covalently cross-linked by MBA to form a stretchable bearing network. In addition, Chai et al. [51] employed extrusion-based 3D printing to fabricate a conductive composite hydrogel dressing with a dual-network structure formed through combined physical and chemical cross-linking. The hydrogels was prepared by blending polyvinyl alcohol (PVA) with κ-carrageenan, adding the conducting polymer PEDOT: PSS, and mesoporous zinc oxide loaded with (+) -catechin (CmZnO). In this system, κ-carrageenan establishes the first network via thermally reversible physical cross-linking, while PVA forms the second network through hydrogen bonding or chemical cross-linking. The dual-network design enhances the material’s mechanical properties, conductivity, and biological activity, and significantly improves the healing rate of infected wounds.

#### 2.3.3. Fully Physically Crosslinked DN Hydrogels

Owing to their limited self-healing ability, fully chemically cross-linked dual-network (DN) hydrogels exhibit poorly adaptable to the dynamic wound environment. In contrast, The physico-chemical hybrid dual network hydrogels integrate reversible physical interactions within a permanent chemical network. The design maintains high strength while providing partial self-recovery and environmental responsiveness, thereby substantially improving the service reliability of the dressing in the complex microenvironment of the wound [52]. Furthermore, fully physical DN hydrogels constructed entirely from dynamic non-covalent interactions, such as multiple hydrogen bonds, host-guest recognition, and coordination bonds, which achieve excellent self-restorability, mechanical strength, and biocompatibility. This progress has significantly broadened the scope of hydrogel research and application [44].

Xia et al. [53] prepared a double-physical cross-linked DN hydrogel (HPAAm-HLPs/Alginate-Ca^2+^) using core-shell hybrid nanoparticles cross-linked polyacrylamide (PAAm) as the first network and Ca^2+^ ion-crosslinked sodium alginate as the second network. In this system, the first network dissipates energy through hydrophobic interactions, while the second network provides elastic recovery and conductivity via reversible ionic cross-linking. This structure endows the hydrogel with high strength, toughness, rapid self-recovery, and excellent fatigue resistance. The hydrogel has been successfully applied in wearable strain sensors, enabling high-sensitivity monitoring of human joint movement as well as subtle physiological activities. Zhou et al. [54] constructed a triple physically cross-linked DN ion-conducting hydrogel (κ-CG/P (AAm-co-AAc)-Fe^3+^) by combining a first network of κ-carrageenan double helix cross-links with a second network that incorporates both hydrophobic cross-linking via Pluronic F127 micelles and coordination crosslinking through Fe^3+^-COO bonds. Their study focused on the mechanism of synergistic enhancement of multiple reversible physical crosslinking: helical structure, hydrophobic interaction, and metal coordination, and excellent fatigue resistance and rapid self-recovery properties. The hydrogel shows significant potential for electronic skin, implantable sensing, and facial expression recognition.

Strategies including physical cross-linking, chemical cross-linking, and double-network architectures provide diverse design dimensions for smart hydrogel dressings. These approaches not only define the fundamental mechanical properties and stability of the hydrogels but also directly confer critical functional features, such as self-healing, environmental responsiveness, and high toughness—required to meet the complex demands of dynamic wound environments. The relationship between self-healing performance and the underlying design principles for different cross-linking mechanisms is systematically summarized in Table 1.

## 3. Composite Hydrogel Material System

In constructing composite hydrogels for intelligent wound monitoring, the selection of materials and their critically determine the overall performance of the final dressing. According to the material components, these systems can be categorized as follows (Table 2). Each category employs distinct interaction mechanisms to achieve sensing, conductivity, and therapeutic functions.

### 3.1. Conductive Polymer-Based Composite Hydrogels

Conductive and biocompatible polymers can be combined to construct intelligent hydrogels with conductivity, biocompatibility and stimulus-responsive properties [60]. Conductive polymers have attracted much attention in the field of developing hydrogels due to their good biocompatibility, flexibility, and mechanical and electrical properties [61]. Polyaniline (PANI), polypyrrole (PPy), and poly (3,4-ethylenedioxythiophene) (PEDOT) can be selected. These polymers possess conjugated π-electron systems, which confer intrinsic conductivity approaching that of conductive materials [62,63]. Natural or synthetic polymers serve as the supporting matrix, balancing mechanical strength and biocompatibilit [64].

#### 3.1.1. PANI

In PANI-based composite hydrogels, the ratio of conductive polymer to polymer matrix is critical for tailoring their overall performance. Devi et al. [65] prepared a series of composite hydrogels with adjustable properties by varying the PANI:PVA ratio. They found that as the PANI content increased, the hydrogel’s conductivity improved, but its crystallinity and swelling rate decreased. Among the formulations, the P6 samples with a specific PANI:PVA ratios exhibited improved conductivity while retaining a relatively high swell rate. The materials were successfully applied in supercapacitors and glucose sensors, demonstrating that an optimal balance between gelation and conductivity can be achieved through fine component design. PANI-based composite hydrogels are not restricted to their original swelling state, and an important trend is to explore the unique properties they exhibit in stable solid forms after drying or post-processing. For instance, Honciuc et al. [66] obtained rubbery elastomers by performing in-situ polymerization of PANI within PVA/glycerol hydrogels followed by a 36 days drying process. The dried material exhibited both conductivity and viscoelastic behavior, with resistance changes under compression recovering slowly. This indicates that after water removal, electrical conduction is governed by the viscoelastic dynamics of the matrix, offering a new principle for designing sensors with tailored response dynamics. In summary, by regulating both the composition and the physical state of the matrix, the electro-chemo-mechanical coupling in such materials can be customized to address diverse monitoring needs in chronic wound care.

#### 3.1.2. PPy

Although PPy exhibits good electrical conductivity, its rigid conjugated backbone tends to cause molecular aggregation, which impairs its uniform dispersion and signal transmission efficiency in a hydrogel matrix. To address this limitation, an effective strategy to use natural polymers rich in anionic groups as “soft templates” to guide the orderly growth of PPy. For instance, Yin et al. [67] prepared PPy/SA composite hydrogels with a three-dimensional nano-network structure via in-situ polymerization. Carboxyl groups on the sodium alginate (SA) chains bind tightly to PPy chains through electrostatic interactions and hydrogen bonds, effectively inhibiting the disordered agglomeration of PPy. Notably, when SA was introduced as a dopant, the composite formed a uniform nanofiber architecture, which significantly reduced the charge-transfer resistance. This highly electrochemically active structure provides an important material basis for constructing highly sensitive sensors capable of monitoring the wound microenvironment. For chronic wound management, smart dressings require not only sensing capabilities but also the ability to maintain a moist healing environment over extended periods. However, conventional hydrogel sensors tend to lose water and harden during use, resulting in diminished performance. To address the challenge of performance degradation caused by dehydration in dry environments or structural rigidification in low-temperature environments, Zhang et al. [68] developed a hemicellulose/PPy/PVA composite hydrogel with excellent environmental adaptability. By introducing glycerol to form a water/glycerol binary solvent system, strong hydrogen bond intaractions are established between water and glycerol molecules. This interaction effectively inhibits water evaporation and disrupts the formation of ice lattices. As a result, the hydrogel maintains excellent flexibility and electrical stability even under harsh conditions. After 7 days of storage at room temperature, it still retained high water content and stable conductivity, ensuring the reliability of long-term continuous monitoring. At the same time, it exhibits a high sensitivity piezoresistive response to human motion, enabling accurate detection of large amplitude movements, such as joint bending. This makes it an ideal material program for long-term wound dynamic monitoring and nursing. In summary, whether through the templating effect of natural polymers to optimize the micro-conductive network or via multi-component recombination recombination to enhance environmental stability, these strategies collectively demonstrate that in the design of PPy-based composite hydrogels, realizing the synergistic unification of microstructure ordering and macroscopic performance persistence is the key to constructing high-performance, long-term chronic wound intelligent monitoring systems.

#### 3.1.3. PEDOT

PEDOT is a conductive polymer known for its favorable electrical conductivity and environmental stability [69], however, it lacks biological activity to promote tissue regeneration. To form an electroactive and bioinductive wound repair interface, combining it with extracellular matrix components is one of the current research directions. Gao et al. [70] designed a dynamically crosslinked conductive hydrogel by integrating PEDOT:PSS into a biomimetic network composed of aminated porcine acellular dermal matrix (APADM), oxidized hyaluronic acid (OHA), and dopamine–iron complexes (DAFe). The resulting gel exhibited good conductivity, deformability, and water absorption. Under electrical stimulation, it significantly accelerated wound healing in a rat model by enhancing angiogenesis and reducing inflammation, demonstrating a viable strategy for developing bioactive and electroresponsive smart dressings. Moreover, the non-degradability of PEDOT limits its suitability for long-term implantable applications. To address this, combining PEDOT with biodegradable natural polymers offers a promising approach. Xu et al. [71] developed a composite based on carboxymethyl chitosan (CMCS) and PEDOT using a two-step strategy to form a semi-interpenetrating network. This composite not only exhibited good conductivity but also showed significant enzymatic degradability, offering a design concept for implantable wound sensors that may avoid the need for later removal. Thus, the biological behavior of PEDOT-based hydrogels can be precisely regulated by synergistically introducing substrates with bioactive or degradable properties, thereby enabling active intervention and non-destructive care of the chronic wound-healing microenvironment while ensuring high-fidelity electrical signal monitoring.

In the design of carbon nanomaterial-based composite hydrogels, the choice of conductive polymers and the strategies for integration plays a decisive role in the material performance. PANI, PPy, and PEDOT are often used to construct intelligent hydrogel systems that integrate conductivity, biocompatibility, and functional responsiveness, owing to their unique π-conjugated structures and tunable electrochemical properties. The core design strategies and key performance characteristics of these three conductive polymers in composite hydrogels are systematically summarized in Table 3.

### 3.2. Carbon Nanomaterial-Based Composite Hydrogels

Carbon nanomaterials, such as include carbon nanotubes (CNTs), graphene oxide (GO), and MXene, are widely used as functional fillers in high-performance intelligent sensing hydrogels due to their excellent intrinsic conductivity, high specific surface area, and good mechanical modulus [72]. Unlike conducting polymers, which rely primarily on electronic transitions within conjugated chains, carbon nanomaterials are covalently incorporated into hydrogel networks. This integration effectively combines the outstanding conductive, mechanical, and optical properties of carbon materials with the three-dimensional hydrophilic biological interfaces of hydrogels [73]. This strategy not only significantly improves the mechanical strength and electrical properties of traditional hydrogels but also imparts the highly sensitive to external stimuli, such as pH, there by opening a new avenue for constructing advenced intelligent monitoring platforms.

#### 3.2.1. CNTs

Carbon nanotubes (CNTs) serve as ideal fillers for conductive composite hydrogels due to their excellent mechanical and electrical properties. However, achieving their uniform dispersion within hydrophilic gel networks remains a key challenge for practical application [74]. Shah et al. [75] employed ultrasound and biocompatible surfactants (BSA) to disperse multiwalled CNTs into a Michael addition cross-linked polyethylene glycol hydrogel. The incorporation of CNTs not only significantly reduced the resistivity of the hydrogel, but also prolonged the gel time and increased the mesh size, owing to their physical hindrance effect. Notably, PEG hydrogels, which originally did not support cell attachment, could act as effective cell anchoring points on the surface of CNT aggregates after the introduction of CNTs, significantly improving the long-term survival rate of encapsulated neural cells. This capability is essential for chronic wound repair and for the further development of smart dressings suitable to complex wound environments. Researchers continue to work toward more integrated, and versatile hydrogel systems. For example, Fan et al. [76] construct a multifunctional organohydrogel by integrating CNTs with nanosilver particles within a dynamic cross-linking network. By partially replacing water with organic solvents (e.g., glycerol or glycol), the freezing point of the solvent system was significantly lowered while volatility was reduced. This design endows the dressing with exceptional environmental stability, maintaining high conductivity and mechanical flexibility even in sub-zero temperatures. Coupled with its excellent self-repair efficiency, broad-spectrum antimicrobial properties, and strong tissue adhesion, this environmental tolerance enables the dressing to function reliably in outdoor or low-temperature environments without failure from freezing or drying. These propertes demonstrate its stability performance in monitoring human movement

However, CNTs still present potential toxicity challenges in biomedical applications, especially in long-term implantation or chronic wound exposure. Studies indicate that CNT toxicity is closely related to their size, aspect ratio, surface chemical properties, dispersion state, and exposure route [77]. For instance, fibrous CNTs may trigger inflammatory responses and fibrosis similar to those of asbestos, while CNTs with insufficiently functionalized surfaces tend to aggregate, causing cell membrane damage and inducing oxidative stress [78]. Therefore, when used in wound dressing, their biological toxicity must be mitigated through surface modification, control of size distribution, and optimization of dispersion strategies, alongside a systematic evaluation of its long-term biosafety.

#### 3.2.2. GO

Graphene oxide (GO) has emerged as a primary nanomaterials for fabricating intelligent composite hydrogel dressings, owing to its unique two-dimensional structure, excellent mechanical properties, good biocompatibility, and abundant surface functional groups that facilitate chemical modification [79]. Tian et al. [80] constructed a multifunctional hydrogel using GO, which served dual roles as a conductive filler for motion sensing and as a photothermal agent for antibacterial therapy. This combined approach demonstrated significant potential for synergistic monitoring and treatment of chronic wounds, with key performance outcomes listed in Table 4. Beyond directly incorporating GO as a functional component, it can also be covalently combined with natural polysaccharides via chemical strategies to construct composite systems with more stable architectures and enhanced bioactivity. For example, Khan et al. [81] developed a composite hydrogel system based on GO-functionalized arabinoxylan for skin wound healing. This chemical bonding strategy yielded a material with excellent antimicrobial properties, biocompatibility, and controlled degradability, effectively promoting skin wound healing, as outlined in Table 3. Such systems not only provide physical support and antibacterial protection for dynamically changing wounds but also offer intrinsic conductivity, modifiability, and more integrated real-time monitoring function. This lays a solid material foundation for next-generation smart wound management.

#### 3.2.3. MXene

MXene is a novel class of carbon nanomaterials with high conductivity and tunable surface chemistry, with great application prospects in biomedicine [82]. Ren et al. [83] developed an injectable composite hydrogel (E-MXene) by complexing MXene nanosheets with a porcine cardiac extracellular matrix hydrogels. This composite actively modulated the pathological microenvironment, promoting tissue repair at the cellular and animal levels, with its core functionalities captured in Table 4. To achieve self-powered and high-sensitivity monitoring in hydrogel dressing, the dispersibility of nanofillers and their impact on the hydrogel’s conductive network are essential. Zhang et al. [84] enhanced the dispersion stability of Ti C Tx MXene via alkali oxidation, introducing TiO nanowires/particles and abundant oxygen-containing functional groups on its surface, which significantly improved the dispersion stability of MXene in hydrogel precursor solutions. They prepared a conductive PAM/OM (POM) hydrogel by complexing oxidized MXene (OM) into a polyacrylamide (PAM) network. The resulting hydrogel exhibited excellent tensile properties and high strain sensitivity, demonstrating MXene’s dual role as both an active therapeutic component and a superior conductive filler for sensitive sensors, as described in Table 4. In summary, MXene can not only be incorporated into hydrogels as active therapeutic components to intervene pathological processes, but also be optimized for dispersibility through surface engineering and used to construct highly sensitive and stretchable sensing networks as excellent conductive fillers.

As representative examples of carbon nanomaterial-based composite hydrogels, Table 4 summarizes typical systems utilizing CNTs, GO, and MXene, along with their design strategies, key properties, and functional features.

**Table 4:** Representative designs of conductive polymer-based composite hydrogels.

Material	Ref.	Core Composite System	Key Design Strategy	Main Performance/Functional Features
CNTs	[75]	CNTs/PEG (with BSA)	Ultrasonic dispersion with biocompatible surfactant (BSA).	Reduced resistivity, prolonged gel time, provided cell attachment sites.
	[76]	CNTs-Ag/Dynamic Network Organohydrogel	Constructing an organic-aqueous hybrid (organohydrogel) system.	Anti-freezing, anti-drying, self-healing (99.67%), antimicrobial, tissue adhesive.
GO	[80]	GO-based Multifunctional Hydrogel	Blending GO as conductive and photothermal agent.	High strain sensitivity, near-infrared photothermal antibacterial (~99.8% healing in 10 days in diabetic model).
	[81]	GO-Arabinoxylan/PVA	Covalent functionalization of GO with arabinoxylan.	Excellent antimicrobial activity, biocompatibility, controlled degradability, promoted healing within 7 days.
MXene	[83]	MXene/Cardiac ECM Hydrogel	Complexing MXene nanosheets with decellularized ECM hydrogel.	Injectable, antioxidant (ROS scavenging), conductive, enhanced intercellular electrical coupling (Cx43↑).
	[84]	Oxidized MXene/PAM	Alkali oxidation of MXene to improve dispersibility.	High conductivity, excellent tensile properties, high strain sensitivity.

### 3.3. Metal Nanomaterials/MOFs-Based Composite Hydrogels

Composite systems incorporating metallic nanomaterials/MOFs into hydrogels merge the advantages of both components. Metallic nanomaterials offer excellent conductivity and electrocatalytic properties [85], while MOFs possess highly ordered porous structures, large specific surface areas, structural flexibility, and good biocompatibility [86]. By embedding these functional components into a biocompatible, three-dimensional hydrogel matrix, a multifunctional integrated platform can be constructed. Such platforms enable realtime electrochemical or optical signal monitoring, deliver on demand antibacterial and anti-inflammatory effects, and facilitate the timely release of growth factors. This integration provides a novel material solution for the intelligent and closed loop management of complex chronic wounds.

#### 3.3.1. Metal Nanomaterial-Based Composite Hydrogels

Due to their unique physicochemical properties, metallic nanoparticles are often incorporated into hydrogel networks to construct composite wound dressings with intelligent response and multifunctional integration. Khodami et al. [87] introduced gold nanoparticles (AuNPs) together with lithium saponite into PNIPA hydrogel networks to construct multifunctional materials with both mechanically enhanced, highly conductive and near-infrared responsive drug release functions. This strategy yielded a composite with significantly enhanced mechanical properties, high conductivity, and a near-infrared-responsive drug release capability, as detailed in Table 5, enabling the combination of strain sensing and on-demand drug administration. In addition to AuNPs, silver nanoparticles (AgNPs) are widely used in smart hydrogels owing to their excellent antibacterial properties and biocompatibility [88]. Xu et al. [89] developed a pH/glucose dual-response photothermal hydrogel loaded with AgNPs with resveratrol (RES). Based on a GelMA/BC double-network backbone, this hydrogel integrates the long-lasting antibacterial activity of AgNPs, the intelligent anti-inflammatory/antioxidant release from RES, and the photothermal effect of polydopamine, synergistically promoting diabetic wound healing (with key outcomes summarized in Table 5). Therefore, the integration of metal nanoparticles into hydrogels not only significantly improve the mechanical and electrical conductivity of materials but also enables environmentally responsive drug release and photothermal synergistic therapy, providing a promising material strategy for intelligent monitoring and precise treatment of chronic wounds.

However, the potential biological toxicity of metal nanoparticles cannot be ignored. Although AgNPs possess broad-spectrum antibacterial properties, the released Ag^+^ ions can penetrate cell membranes, interfere with cellular metabolism, induce ROS generation, and cause DNA damage [90]. AuNPs are relatively inert, but long-term accumulation may still trigger cytotoxicity or immune responses [91]. Moreover, the biocompatibility of metal particles is influenced by their size, morphology, surface charge, and aggregation state [92]. In the design of composite hydrogels, it is necessary to consider the balance between their antibacterial/conductive functions and biosafety.

#### 3.3.2. MOFs Based Composite Hydrogels

The combination of metal-organic frameworks (MOFs) with hydrogels offers a versatile platform for managing chronic wound microenvironments. MOFs can be functionalized by loading metal ions or drugs, while the tructural design of the composite imparts environmental responsiveness and biological regulatory capabilities [93]. Qiu et al. [94] developed a ROS-responsive polyvinyl alcohol (PVA) hydrogel loaded with ZIF-8 nanoparticles to modulate the atopic dermatitis microenvironment. The hydrogel possessed a porous structure and good swelling properties, enabling efficient ROS scavenging and protection of cells from oxidative damageas, as summarized in Table 5, This work provides a combined MOFs-hydrogel strategy for microenvironmentally regulated dressings in chronic inflammatory wounds. Beyond microenvironmental regulation, MOF-hydrogel composites also showed advantages in drug loading and tissue regeneration. For example, Wang et al. [95] developed a photoresponsive composite hydrogel based on copper-niacin MOFs (CuNA) with gelatin methacryloyl (GelMA). This system enabled the controlled release of basic fibroblast growth factor (bFGF) and copper ions, synergistically exerting antibacterial, pro-angiogenic, and pro-healing effects, significantly accelerating wound closure in a full-thickness skin defect model (key features presented in Table 5). Thus, MOF-hydrogel composite systems exhibit multifunctional integration potential in chronic wound management. They can modulate the wound microenvironment through mechanisms such as ion release and ROS clearance, while simultaneously promoting tissue regeneration via the controlled delivery of growth factors.

**Table 5:** Representative designs of metal nanomaterials/MOFs-based composite hydrogels.

Material Type	Ref.	Core Composite System	Key Design Strategy	Performance Features
AuNP	[87]	AuNPs-Laponite/PNIPAm	Incorporating AuNPs and Laponite into thermo-responsive PNIPAm network.	Enhanced tensile strength and conductivity; Enables NIR-triggered drug (doxorubicin) release and strain sensing.
AgNP	[89]	AgNPs-RES/GelMA-BC DN	Loading AgNPs and resveratrol into a GelMA/BC double-network hydrogel.	pH/glucose dual-responsive release; Combines photothermal antibacterial (via PDA) with antioxidant/anti-inflammatory therapy.
ZIF-8	[94]	ZIF-8/PVA Hydrogel	Loading ZIF-8 nanoparticles into a PVA hydrogel matrix.	ROS-scavenging; Sustained Zn^2+^ release for antibacterial action; Modulates inflammatory microenvironment.
CuNA	[95]	CuNA-bFGF/GelMA	Incorporating a CuNA (loaded with bFGF) into GelMA hydrogel.	Photoresponsive; Controlled co-release of Cu^2+^ and bFGF; Antibacterial, pro-angiogenic, and enhances collagen deposition.

### 3.4. Multivariate Material Composite System

Multicomponent composite systems integrate diverse functional materials, such as conductive polymer, carbon nanomaterial, metal nanomaterial, and MOFs, within a hydrogel matrix. For instance, Xu et al. [96] reported a quaternary composite conducting hydrogel system incorporating PANI hydrogel with tannic acid-chelated AgNPs (TA-AgNPs) as well as CNTs. In this design, PANI serves as a sensing substrate for the synchronous electrochemical detection of pH and tyrosine; CNTs enhance electrical conductivity and mechanical stability; and TA-Ag NPs provide antibacterial properties. A key feature of this system its evaluation of the influence of pH on tyrosine detection, coupled with the use of real-time pH measurements to calibrate the readouts, thereby significantly improving detection accuracy in a variable-pH environment. Cheng et al. [59] developed a five-membered composite system by integrating Zn-MOF with MXene using oxidized bacterial cellulose as a matrix. The material showed excellent photothermal response, rapid heating and synergistic release of Zn^2+^ under near-infrared light irradiation, achieving a synergistic bactericidal rate of more than 99% against both *Staphylococcus aureus* and *Escherichia coli*. In vivo experiments further demonstrated that the system effectively cleared infection, modulated inflammation and promoted angiogenesis and collagen deposition, thereby significantly accelerating the healing of wound. This study provides a successful material-integration strategy for designing of intelligent dressings that combine efficient antibacterial and active repair functions. Thus, through synergistic interactions among its components, a multicomponent composite system not only overcomes the performance limitations of individual materials but also establishes a new material paradigm capable of both dynamic monitoring and active treatment. This advancement promotes the evolution of wound dressings toward intelligent, systematic, and multifunctional platforms.

## 4. Multi-Physiological Index Monitoring System for Composite Hydrogels

Real-time dynamic monitoring of key physiological and biochemical parameters is essential for managing the progression and healing of chronic wounds. As three core parameters with clear clinical relevance, pH, local temperature, and interface pressure together form the monitoring basis of an intelligent wound evaluation system [97]. Changes in pH are directly related to the microenvironmental conditions that favor microbial proliferation and biofilm formation, serving as an early indicator of infection risk. Local tissue temperature reflects the metabolic activity of inflammatory cells and provides a reliable thermal signal of the magnitude of the clinical inflammatory response. Interface pressure is a key mechanical variable to realize the early warning of stress injury risk. Real-time dynamic monitoring of physiological parameters is crucial for managing the pathological evolution and healing of chronic wounds. pH, local temperature, and pressure—three well-defined, clinically relevant indicators, serve as the foundation for intelligent wound assessment systems. However, the chronic wound microenvironmen is highly complex and dynamic. Changes in a single parameter are often influenced by multiple factors, rendering it insufficient to provide a comprehensive assessment of wound status [98]. For example, elevated pH levels may indicate bacterial infection, but can also be affected by exudate composition, tissue necrosis, or local metabolic activity. Similarly, localized temperature changes may reflect inflammation, but are also associated with factors such as blood perfusion and ambient temperature. Relying solely on a single parameter thus carries a risk for misinterpretation. A comprehensive and accurate wound assessment requires multi-parameter monitoring, cross-validation, and integration with clinical manifestations.

**Figure 2:**
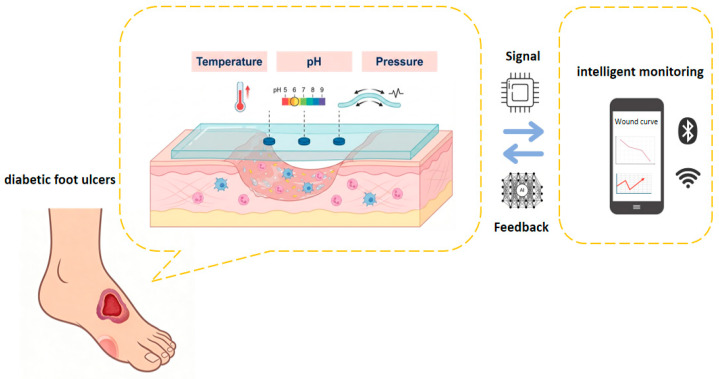
The hydrogel dressing on the wound surface monitors chemical, thermal, and mechanical signals, which are transmitted to the network.

Due to its modular chemical function design, tunable thermal conduction/response properties, and adaptive mechanical network structure, the composite hydrogel material system offers a unique materiological solution for integrating multimodal sensing units. It can convert chemical, thermal, and mechanical signals for the wound microenvironment into related datasets through a material-tissue interface coupling mechanism. These datasets support an intelligent diagnosis and treatment decision-making system based on physiological feedback to, enabling precise regulation throughout the full-cycle management of chronic wounds (Figure 2).

### 4.1. pH

pH is a critical indicator of infection status and tissue regenerating in chronic wound healing [99]. The healthy human skin typically maintains a pH range of 4.5 to 5.5. The physiological processes of wound healing is complex and dynamic, progressing through hemostatic, inflammatory, proliferative, and remodeling phases [100]. In acute wounds, pH is around 6.2 during hemostasis, becomes more acidic in the inflammatory phase, gradually rises during granulation, and finally returns to normal levels upon epithelial reformation [101]. In contrast, chronic wounds often exhibit an alkaline pH between 7 and 9, which favors bacterial infection [102]. Therefore, accurate, timely, and early detection of infection and real-time monitoring of infection-related biomarkers are critical for the effective treatment and management of chronic wounds. [103].

A common method for monitoring pH changes is colorimetric sensing, which relies on indicator molecules whose optical properties reversibly change with pH. These spectral change typically result from structural alterations in the indicator molecules upon protonation or deprotonation, leading to characteristic shifts or intensity variations in the UV-Vis absorption or fluorescence emission spectra [104]. Based on this principle, researchers have developed varous smart wound dressings. For example, Yao et al. [105] fabricated a nanofibrous dressing incorporating starch-derived carbon quantum dots (CQDs). This dressing exhibited reversible color and fluorescence changes across a pH range of 5 to 8. By capturing images with a smartphone and performing RGB analysis, quantitative monitoring of wound pH could be achieved. Furthermore, such colorimetric sensing has been combined with active treatment capabilities. Zou et al. [106] developed an intelligent hydrogel based on bimetallic FeNi-MOF nanozymes and phenol red. This system not only anables real-time, smartphone-readable pH monitoring but also utilizes the cascade catalytic activity of the MOFs nanozymes to generate antibacterial ROS from wound hydrogen peroxide, enabling combined diagnosis and targeted therapy. Therefore, colorimetric methods offer in an intuitive and low-cost manner for real-time wound pH monitoring, and when combined with novel nanomaterials or enzyme-catalyzed modules, providing important ideas for the development of the next generation of chronic wound management platforms with intelligent diagnosis and active intervention capabilities.

Compared with direct colorimetric methods, the potentiometric method relies on the Nernst equation in electrochemistry, using a sensitive material with a selective response to H^+^ ions to measure the potential difference between the working and reference electrode. This potential difference exhibits a logarithmic relationship with pH, enabling continuous, high-precision electrical signal output. However, when potentiometric sensors are placed in complex wound exudates, adsorption of proteins, cells, and bacteria can cause severe biofouling, leading to signal driff, reducing sensitivity, and shortened device lifespan. To address this critical challenge, Zhang et al. [107] designed a flexible potentiometric sensor chip based on a self-adhesive poly (sulfobetaine methacrylate) -dopamine-polyethylene glycol diacrylate hydrogel coating. The hydrogel coating exhibits low swelling, good biocompatibility, and high tensile strength, allowing it to firmly adhere to all sensor surfaces. This provides consistent and long-lasting anti-fouling protection for the entire electrode system. This work not only confirms the high reliability of potentiometry in wound monitoring but also provides a universal solution for developing long-term, stable, interference-resistant wearable potential-sensing systems through material interface engineering.

### 4.2. Temperature

Temperature is an key physiological parameter in the chronic wound healing, serving as an indicator of metabolic activity, inflammatory status, and blood perfusion [108]. During the inflammatory phase or in the presence of infection, metabolic activity and increased blood flow typically raise the local temperature of the wound [109]. Conversely, as healing progresses into the proliferative or remodeling phase, blood supply and metabolism stabilize, and temperature gradually returns to near normal tissue levels [110]. Therefor, continuous and precise monitoring of wound surface and surrounding temperatures can help indicate infection risk, assess inflammation severity, and predict healing progression.

Develop a flexible sensing substrate with intrinsic temperature sensitivity presents a key material challenge for smart wound dressings that must balance biocompatibility with precise monitoring capabilities. Pang et al. [111] reported an ionic conductive thermo-responsive hydrogel in which a poly (N-isopropylacrylamide-co-acrylamide) network was combined with a tannic acid/Fe^3+^ coordination network. This design successfully tuned the volumetric phase transition temperature of the material to a range near the physiological environment of the wound. Although primarily explored for general wearable health monitoring, the hydrogel demonstrated high tensile strength, strong adhesion, and stable temperature-electrical response, suggesting its suitability for integration into dedicated wound-management systems. In a more wound-focused approach, Jiang et al. [112] designed a temperature responsive composite hydrogel dressing based on poly (N-isopropylacrylamide). The innovation is the utilization of PAA-g-PNIPAM as a thermosensitive matrix, whose volume changes reversibly near a lower critical solution temperature (LCST) close body temperature. In addition, silver nanowires are introduced to form a three-dimensional conductive networks, converting the volume change of the thermo-responsive matrix into a measurable change in resistance. This ingenious design effectively transforms the hydrogel itself onto a highly sensitive temperature sensor.

### 4.3. Pressure

The healing of chronic wounds involves dynamic changes in tissue mechanical properties, and the normal healing of the wound is influenced by a balance between external stresses and internal strains [113]. During the inflammatory phase, tissue edema and inflammatory exudation increase local pressure, reducing blood circulation and oxygen supply [114]. In the proliferative phase, fibroblasts proliferation in the subcutaneous tissue and the contraction of newly formed granulation tissue alter the internal stress distribution [115]. Throughout the remodeling phase, collagen reorganization is particularly sensitive to the mechanical environment, and moderate continuous pressure help improve scar texture and color [116].

To address the requirements for pressure monitoring in chronic wounds, researchers strive to improve the piezoresistive sensitivity of hydrogels while simultaneously coordinating design material and structural design to unify monitoring performance with the wound-healing microenvironment. For instance, Li et al. [117] constructed a physical crosslinking network of polyvinyl alcohol (PVA) with an acrylamide-ionic liquid (AM-IL) chemical polymerization network to construct a poly (vinyl alcohol)/acrylamide-ionic liquid (PAIL) composite hydrogel dressing with ionic conductivity. The dressing exhibits a pressure sensitivity of up to 9.19 kPa^−1^ and can stably monitor real-time signal variations from different body regions during movement or compression. Wang et al. [118] developed a textile composite hydrogel dressing by incorporating polydopamine-silver coated calcium phosphate nanoparticles (CPO@PDA-Ag) and vascular endothelial growth factor. This design yields a multifunctional dressing that combines antibacterial activity, breathability, pro-healing and pressure-sensing capability. This structure-function integration design offers a novel strategy for chronic wound pressure monitoring and contributes to the prevention of pressure-induced ulcers. Overall, the design of composite hydrogels is developing towards high sensitivity, multifunctionality, and biomimetic adaptation. By deeply aligning pressure sensing mechanisms with the physiological demands of wound healing, these smart dressings are expected to provide a more precise and integrated solution for chronic wound management.

To accommodate the demands of long-term wear and dynamic stress environments, recent research has focused on enhancing the mechanical durability of pressure-sensing hydrogels [119]. By constructing dual-network structures, incorporating elastic nanofibers, or employing gradient structural designs, it is possible to significantly enhance the material’s fatigue resistance, self-recovery capability, and interfacial stability—without compromising high sensitivity, thereby extending the operational lifespan of the sensor. For instance, Xu et al. [120] developed a bio-derived hydrogel (C-G-P) with an interpenetrating network structure, fabricated from chitosan-gelatin composites and sodium phytate via electrostatic crosslinking, using a gelation-soaking method. In this hydrogel, the two networks are interconnected via shared crosslinking points, enabling uniform stress distribution and synergistic dissipation throughout the system. This results in significantly enhanced modulus and toughness under both compression and tension. Furthermore, owing to the reversibility of its physically crosslinked network, the hydrogel demonstrates excellent self-recovery and fatigue resistance under cyclic loading, making it highly suitable for wearable pressure monitoring in long-term dynamic stress scenarios.

As shown in Table 6, pH, temperature and pressure represent three core physiological signals. Their monitoring methods, technical characteristics and applicable scenarios have distinct emphases. The sensing design of composite hydrogel provides a common platform for integrating these monitoring functions into smart dressings.

## 5. Application of Intelligent Monitoring in Typical Chronic Wounds

This review systematically examines various composite hydrogels developed for intelligent wound monitoring, encompassing their fundamental design strategies, multifunctional material systems, and sensing mechanisms for key physiological indicators such as pH, temperature, and pressure. The advancement of these materials is ultimately directed toward solving practical clinical problems. Considering the diversity of chronic wound types and the varying underlying pathological microenvironments, the functional requirements for smart dressings must be specifically tailored. This chapter will focus on diabetic foot ulcer (DFU) and pressure injuries (PI), two representative chronic wound types with prominent clinical challenges. Through the analysis of DFU and PI, this sectionaims to illustrate the main pathways and core principles for translating intelligent composite hydrogel from the laboratory to clinical application.

### 5.1. DFU

DFU is one of the most serious chronic complications of diabetes. It manifests as a full-thickness skin defect caused by a combination of diabetic neuropathy, peripheral vascular disease, and foot biomechanical abnormalities [121].The DFU microenvironment is high complex, shaoed by endogenous alterations, such as neurological, vascular, immune, and metabolic alterations, combined with exogenous factors, such as infection and trauma, lead to the development of DFU, which is characterized by hyperglycemia, ischemia, hypoxia, excessive inflammation, and persistent infection [122]. Consequently, DFUs exhibit delayed healing and a high recurrence rate. Traditional dressings are difficult to cope with the multiple challenges mentioned. Therefore, developing composite hydrogel dressings that combine environmental responsiveness, mechanical adaptability and multifunctional synergy has become an important research direction in DFU management.

To address the challenges, researchers have developed various composite hydrogel dressings for the effective management of DFUs. Liang et al. [123] designed a pH/glucose dual-responsive hydrogel based on dynamic Schiff base and phenylborate bonds, which exhibited self-healing, tissue adhesion, and mechanical adaptation, enabling intelligent drug release in response to wound conditions. To cope with the extreme temperature and humidity associated with DFUs, Liu et al. [124] developed a double-network composite hydrogel (G-PAGL) employing a water/glycerol binary solvent system, granting it a wide operational temperature range and stability in harsh environments. In addition to sensorized composite hydrogel dressings, functional composite hydrogels have also shown important potential in DFU treatment. Shi et al. [125] constructed a multifunctional hydrogel by combining a polydopamine-polyethyleneimine (PDA-PEI) complex with GelMA. This system leverages the adhesive and antioxidant properties of PDA, the cationic antibacterial effect of PEI, and the biocompatibility of GelMA to synergistically modulate the diabetic wound microenvironment and promote healing. These studies demonstrate that composite hydrogel dressings can intervene in DFU healing at multiple levels, representing a key direction for advanced wound care materials.

### 5.2. PI

PI, commonly referred to as pressure ulcers or bedsores in clinical practice, is localized damages to the skin and underlying doft tissue that occur over a bony prominence as a result of prolonged pressure, shear, or friction [126]. Pressure causes obstruction of the skin microcirculation, leading to local tissue ischemia, hypoxia, and insufficient nutrient supply, and ultimately to skin tissue necrosis. When blood flow is restored following ischemia, a surge in reactive oxygen species (ROS) contributes to ischemia-reperfusion injury, causing secondary damage and further hindering wound healing [127]. An intelligent monitoring system based on composite hydrogel provides a precise, objective, and continuous technical solution for the prevention and management of PI.

Owing to their excellent customizability and multifunctional integration ability, composite hydrogels hold unique value in the intelligent monitoring and treatment of pressue injuries. By incorporating conductive media or drug-loaded nanoscale units, traditional passive dressings can be andowed with real-time sensing and responsiveness, offering novel strategies for the dynamic management of chronic wounds. For instance, Li et al. [117] developed a PVA/acrylamide-ionic liquid composite hydrogel that combines broad-spectrum antibacterial activity with high-sensitivity pressure sensing. This enables real-time monitoring of pressure in bedridden patients and accelerating wound healing. In a study of drug-loaded hydrogel dressings against infectious stress injury, Choipang et al. [128] engineered a sustained-release antimicrobial system by loading PLGA/ciprofloxacin nanoparticles into a PVA hydrogel via γ-radiation crosslinking, which provided prolonged drug release and effective antibacterial action. Look forward, integrating multimodality sensors with feedback controlled drug release system could enable the construction of closed-loop regulated intelligent hydrogel dressings. Such systems are expected to achieve real-time monitoring and adaptive intervention of PI microenvironment, thereby significantly improving healing quality of chronic wounds and clinical nursing efficiency.

The studies summarized above and in Table 5 illustrate the tailored application of smart composite hydrogels in managing specific chronic wounds. A conceptual overview of this material-driven, wound-specific therapeutic strategy is presented in Table 7. The schematic outlines the intelligent wound management cycle enabled by composite hydrogels. From sensing key wound parameters and transducing these signals to data processing and subsequent feedback actions. By integrating sensing, diagnosis, and treatment into a single platform, composite hydrogels bridge the critical gap between passive wound coverage and active, closed-loop management, paving the way for personalized, adaptive therapies for complex chronic wounds such as DFU and PI.

## 6. Summary and Outlook

Intelligent monitoring of chronic wounds represents an advancing frontier at the intersection of biomedicine and flexible materials science. This review has examined the progress in composite hydrogel wound dressings designed for intelligent monitoring. By integrating conductive fillers, including conductive polymers, carbon nanomaterials, and metal nanomaterials/MOFs—into biocompatible hydrogel networks through physical, chemical, and dual-network cross-linking strategies, researchers have successfully constructed a series of novel materials. These composites exhibit excellent mechanical properties, environmental adaptability, and biofunctionality. These composite hydrogel dressings enable intelligent monitoring of key wound healing indicators, such as pH, temperature, pressure, etc., transform biological signals into recordable electrical or optical signals. They also offer antibacterial, anti-inflammatory, and drug-loading capabilities, allowing active intervention in the wound microenvironment to promote the healing of chronic wounds like DFU and PI. From single-functional materials to multi-element composite architectures, and from passive coverage to intelligent monitoring, the application of composite hydrogel dressings in chronic wound care is evolving toward greater precision, versatility and intelligence.

Despite significant advance in intelligent monitoring, conductive hydrogel dressings still face both opportunities and challenges in realistic clinical translation. Tackling these hurdles will be pivotal for translating laboratory innovations into reliable, clinically viable solutions and for advancing the field of composite hydrogel dressings. First, ensuring the long-term stability and biosafety of dressing materials in the complex, dynamic wound environment is paramount. It is essential to assess the long-term chemical stability, leaching profiles, and potential migration of both the hydrogel network and functional fillers in biologically relevant media that simulate wound exudate. Systematic investigation is needed into the release kinetics of fillers from the gel matrix and their potential local and systemic toxicity. Strategies such as surface modification and covalent immobilization should be leveraged to minimize biosafety risks. Second, mechanical durability and sensing reliability are crucial. Dressings must endure cyclic tensile, compressive, and shear stresses resulting from daily activities such as joint movement and muscle contraction. Therefore, hydrogel sensing units require rigorous cyclic-loading and fatigue-recovery testing to evaluate the reproducibility, hysteresis, and long-term drift of electrical (e.g., resistance, capacitance) or optical responses. Developing dynamic network architectures with excellent fatigue resistance and rapid self-recovery is key to ensuring long-term signal reliability. Third, achieving high sensing specificity and strong anti-interference capability in complex biological media remains a significant challenge, as dynamic changes in the wound microenvironment can significantly perturb sensor signals. In addition, current research focuses on the monitoring of single parameters, such as pH or temperature. However, a single index is insufficient for a comprehensive assessment. Crucially, research on artificial intelligence and machine learning algorithms required to fuse these multi-parameter data (pH, temperature, and pressure) into accurate clinical staging remains critically insufficient. This lack of advanced data processing capability acts as a major bottleneck, limiting the transition from laboratory data acquisition to active, precise clinical treatment. Therefore, future smart dressings must not only in multi-dimensional sensing hardware but also in the development of robust algorithms. By training models to identify the unique multi-parameter signatures of different healing stages, the system can achieve accurate staging and dynamic tracking, ultimately enabling the leap from raw data to actionable clinical decisions. Composite hydrogels offer a promising avenue for intelligent monitoring of chronic wounds. Moving forward, increase efforts focus on overcoming the practical challenges of clinical translation and advancing material design from functional display to system integration and clinical application. With continued progress in science and technology, smarter and personalized composite hydrogel dressing for chronic wound care will undoubtedly emerge. Such advances are expected to fundamentally improving patients’ quality of life and drive a transformative shift in the field of wound management.

## Figures and Tables

**Table 1:** Self-healing mechanisms and properties of composite hydrogels based on different crosslinking strategies.

Crosslinking Strategy	Ref.	Core Mechanism & Material Design	Self-Healing Performance	Key Characteristics
Physical Crosslinking	[26]	Host-guest interaction (carborane-β-cyclodextrin).	Rapid healing within 15 min at room temperature.	Provides dynamic reversibility and injectability to fit irregular wounds.
	[28]	Hydrogen bonding between polyethylene polyamine (PPA) and gelatin.	Exhibits thermo-reversible self-healing ability.	Confers pH-responsive behavior(collapses under acidic conditions).
Chemical Crosslinking	[39]	Click chemistry forming disulfide bonds.	Redox-responsive degradation enables on-demand repair/drug release.	Enables construction of intelligent drug delivery systems with controlled release.
Physico-Chemical DN	[50]	First network: Physical crosslinking (electrostatic/H-bond); Second network: Chemical crosslinking (MBA).	Maintains high strength (3.33 MPa) and toughness (1106% strain) with recoverable network.	Provides anisotropic conductivity (13.08 S/m) for flexible multi-functional sensors.
	[51]	First network: Thermally reversible κ-carrageenan; Second network: PVA via H-bond/chemical crosslinking.	Integrated design enhances mechanical properties and bioactivity.	Combines conductivity (PEDOT:PSS) with antibacterial activity (ZnO-catechin), accelerating wound healing.
Fully Physical DN	[53]	First network: Hydrophobic interaction (core-shell nanoparticles); Second network: Ionic crosslinking (Ca^2+^-alginate)	Exhibits high toughness, rapid self-recovery, and excellent fatigue resistance.	Suitable for wearable strain sensors with high sensitivity for monitoring physiological activities.
	[54]	Triple physical crosslinking: helix (κ-carrageenan), hydrophobic (Pluronic F127), coordination (Fe^3+^-COO).	Excellent fatigue resistance and rapid self-recovery (high toughness: 9.82 MJ/m^3^).	High ionic conductivity and ultra-high sensitivity for pressure sensor arrays (<200 Pa).

**Table 2:** Comparison of Material Systems and Functional Properties of Composite Hydrogel Dressings.

Composite System	Typical Materials	Functional Characteristics	Advantage	Limitations/Challenges	Reference
Conductive Polymer-Based Composite Hydrogels	PANI, PPy, PEDOT	Intrinsic conductivity, redox activity, certain biocompatibility	Adjustable conductivity, easy polymerization/recombination, relatively low cost	Insufficient environmental stability, poor biodegradability of some polymers, brittle mechanical properties	[55]
Carbon nanomaterial-based composite hydrogels	CNTs, GO, MXene	As a biomimetic scaffold, it provides three-dimensional porous structure, supports cell adhesion, proliferation and differentiation, and can achieve intelligent response through light, heat, electricity and other stimuli	Excellent biocompatibility, adjustable mechanical properties, conductivity and drug-loading capacity, suitable for a variety of tissue repair	Insufficient mechanical strength, potential biological toxicities (particularly CNTs), complex preparation process, unknown long-term safety in vivo	[56]
Metal Nanomaterials Composite Hydrogels	AgNPs, AuNPs	It also has multifunctional integration characteristics such as mechanical enhancement, environmental response, antibacterial, healing promotion and drug controlled release.	Combined with the biocompatibility of hydrogels and the efficient function of metal nanoparticles, synergistic antibacterial, intelligent response, long-acting sustained release and tissue repair are realized.	High cost (e.g., Au), potential metal ion release toxicity, agglomeration inactivation	[57]
MOFs Composite Hydrogels	ZIF-8, MIL et al.	Antimicrobial and anti-inflammatory effects, stimulation of responsive drug release, promotion of tissue regeneration and angiogenesis	Multifunctional integrated platform, biocompatibility and degradability, high drug loading capacity and controlled release capacity	Readily degradable in aqueous environment, potential biotoxicity, scale production and cost challenges	[58]
Multivariate material composite system	Combination of the above materials	Excellent mechanical properties, antimicrobial activity and cytocompatibility	Break through the limitation of single material and realize the synergistic effect of “1 + 1 > 2”	The preparation process is more complex and the interfacial interactions between components require fine regulation	[59]

**Table 3:** Representative designs of carbon nanomaterial-based composite hydrogels.

Conductive Polymer	Ref.	Core Composite System	Key Strategies	Main Performance Characteristics
PANI	[65]	PANI/PVA	Precise control of the ratio between conductive polymers and the matrix.	Achieve adjustable and balanced conductivity, swelling properties, and gelation degree.
	[66]	PANI/PVA/glycerol	After in situ polymerization, dehydration occurs to form an elastomer.	Electrical conductivity is deeply coupled with the viscoelasticity of the matrix, and the change in electrical resistance follows a viscoelastic relaxation model.
PPy	[67]	PPy/Sodium alginate	Using natural polyanions (SA) as “soft templates” to guide in situ polymerization.	Effectively suppresses PPy aggregation, forming a three-dimensional conductive nanonetwork that reduces charge transfer resistance.
	[68]	hemicellulose/PPy/PVA	Glycerol was introduced to establish a binary solvent system.	Significantly enhances the water retention, environmental stability, and long-term flexibility of hydrogels.
PEDOT	[70]	PEDOT:PSS/APADM-OHA-DAFe	Dynamically composites with decellularized dermal matrix and hyaluronic acid biomimetic networks.	Combines high electrical conductivity (0.2–0.3 S/m) with biological activity; promotes angiogenesis and anti-inflammatory effects under electrical stimulation.
	[71]	PEDOT/Carboxymethyl chitosan	Constructing a semi-interpenetrating conductive network within a biodegradable CMCS network.	Possesses excellent electrical conductivity and enzymatic degradability (approximately 35% degradation within 10 weeks).

**Table 6:** Monitoring method of multiple physiological indexes of composite hydrogel.

Monitoring Signal	Monitoring Method	Principle	Characteristic
pH	Colorimetry	Protonation/deprotonation of the indicator molecule results in changes in its absorption or emission spectra that cause changes in color or fluorescence that are visible to the naked eye [104].	Intuitive, low cost, no complex circuit, easy to combine with smartphone to realize semi-quantitative analysis [105].
	potentiometric method	Following the Nernst equation, this potential difference is related to the logarithm of pH [107].	High precision, continuous real-time monitoring, stable output of electrical signals, easy to integrate.
temperature	resistivity/thermal method	The thermal signal is converted into electrical signal through a conductive network using the property that the resistance or volume of a temperature sensitive material changes with temperature.	Continuous monitoring, high sensitivity and remote transmission [111].
pressure	piezoresistive method	Hydrogels deform when compressed causing changes in density or contact resistance of internal conductive pathways causing resistance changes [117].	Interface pressure associated with PI can be directly monitored, as well as changes in internal stress caused by granulation tissue growth, edema [117].

**Table 7:** Representative composite hydrogel designs for managing chronic wounds (DFU and PI).

Chronic Wound Type	Ref.	Core Composite System/Strategy	Key Functional Features	Performance Highlights/Application Outcome
DFU	[123]	pH/glucose dual-responsive hydrogel based on Schiff base & phenylborate dynamic bonds.	Self-healing, tissue adhesion, mechanical adaptation; On-demand release of metformin triggered by wound pH/glucose.	Significantly accelerated DFU healing in responsive microenvironment.
	[124]	DN hydrogel (G-PAGL) based on polyacrylamide/gelatin/ε-polylysine with water/glycerol binary solvent.	Broad environmental tolerance (−20 to 60 °C), anti-freezing, anti-dehydration, maintains mechanical & adhesive properties in harsh conditions.	Adapted to foot movement pressures; Promoted DFU healing under extreme temperature/humidity.
	[125]	Multifunctional composite hydrogel of PDA-PEI complex with photo-crosslinked GelMA.	Self-healing, high tensile strength, strong adhesion; Combines PDA’s adhesion/antioxidant, PEI’s cationic antibacterial, and GelMA’s biocompatibility.	Promoted angiogenesis, collagen deposition, macrophage phenotype switching; Accelerated healing in MRSA-infected diabetic mouse model.
PI	[117]	PVA/acrylamide-ionic liquid composite hydrogel via copolymerization & freeze-thaw cycling.	Broad-spectrum antibacterial activity; High-sensitivity pressure sensing.	Sensitivity ~9.19 kPa^−1^; Enabled real-time monitoring of patient movement pressure; Accelerated full-thickness wound healing by inhibiting infection and promoting regeneration.
	[128]	PVA hydrogel loaded with PLGA/ciprofloxacin nanoparticles via γ-radiation crosslinking.	Sustained antimicrobial drug release; Nano-micro composite structure; Good biocompatibility.	Sustained drug release up to 4 days; Effective inhibition against *E. coli* and *S. aureus*.

## Data Availability

The original contributions presented in this study are included in the article. Further inquiries can be directed to the corresponding author.
